# An Unusual Presentation of Dermatomyositis With Muscle Hypertrophy

**DOI:** 10.7759/cureus.41005

**Published:** 2023-06-26

**Authors:** George Bailey, Jaya R Trivedi

**Affiliations:** 1 Neurology, University of Texas (UT) Southwestern Medical Center, Dallas, USA

**Keywords:** muscle hypertrophy, malignancy, isaacs syndrome, neuromyotonia, dermatomyositis

## Abstract

Peripheral nerve hyperexcitability is a rare disorder characterized by spontaneous motor unit activity. Although peripheral nerve hyperexcitability is seen in multiple immune-mediated neurological conditions, an association with dermatomyositis has rarely been reported. We present a 65-year-old woman with serological and muscle biopsy features of dermatomyositis who also developed marked muscle hypertrophy, stiffness, and delayed relaxation along with electrodiagnostic features of peripheral nerve hyperexcitability such as that seen in Isaacs syndrome.

## Introduction

Peripheral nerve hyperexcitability (PNH) is a rare disorder characterized by continuous spontaneous motor unit activity resulting in increased muscle activation. Clinically, this can manifest as cramps, stiffness, delayed relaxation, fasciculations, and myokymia, along with extramuscular symptoms such as diarrhea and sweating. This spontaneous activity persists during sleep and general anesthesia. Nerve conduction can show after-discharges, and electromyography can reveal spontaneous motor unit activity, including fasciculation potentials, myokymia, and neuromyotonic discharges.

There are both hereditary and acquired forms of peripheral nerve hyperexcitability. Isaacs syndrome is a well-known acquired disorder of PNH characterized clinically by myokymia, twitching, muscular hypertrophy, and sweating. It is thought to be an immune-mediated phenomenon. Thirty-eight percent to 50% of cases have autoantibodies against voltage-gated potassium channel (VGKC) complex proteins [1,2], including antibodies against leucine-rich glioma inactivated protein 1 (LGI-1) and contactin-associated protein 2 (CASPR2) [3,4].

Although PNH has been associated with multiple other autoimmune disorders, including myasthenia gravis, Guillain-Barre syndrome, and chronic inflammatory demyelinating polyneuropathy [5], it has rarely been reported in conjunction with dermatomyositis. We present a 65-year-old woman with serological and muscle biopsy features of dermatomyositis who also developed clinical and electrodiagnostic features of Isaacs syndrome.

This article was previously presented as a meeting abstract at the 2023 Carrell Krusen annual neuromuscular symposium on February 24, 2023.

## Case presentation

A 65-year-old woman with a history of hypothyroidism presented with a heliotrope and shawl-like rash. Two months later, she developed proximal lower extremity muscle weakness as well as stiffness and reduced range of motion in the left arm. Serum creatine kinase was elevated at 4068 U/L. Needle electromyography revealed a myopathic process. A myositis antibody panel was positive for Mi2-α (38; reference level < 11 SI) and Mi2-β (23; reference level < 11 SI) antibodies. Anti-signal recognition particle (anti-SRP) and anti-3-hydroxy-3-methylglutaryl-coenzyme A reductase (anti-HMGCR) antibodies were negative. A muscle biopsy of the left biceps showed inflammatory myopathy with multifocal lymphocytic inflammatory infiltrates, perifascicular atrophy, and occasional necrotic myofibers. Immunohistochemistry showed robust expression of MHC class I and deposition of C5b-9.

The patient was diagnosed with dermatomyositis and treated with oral prednisone up to 60 mg/day. Malignancy investigations, including a CT scan of the chest, abdomen, and pelvis, demonstrated a possible peritoneal mass. Mammography was negative. A subsequent PET scan showed diffuse skeletal muscle uptake consistent with myositis but was negative for an underlying malignancy.

**Figure 1:**
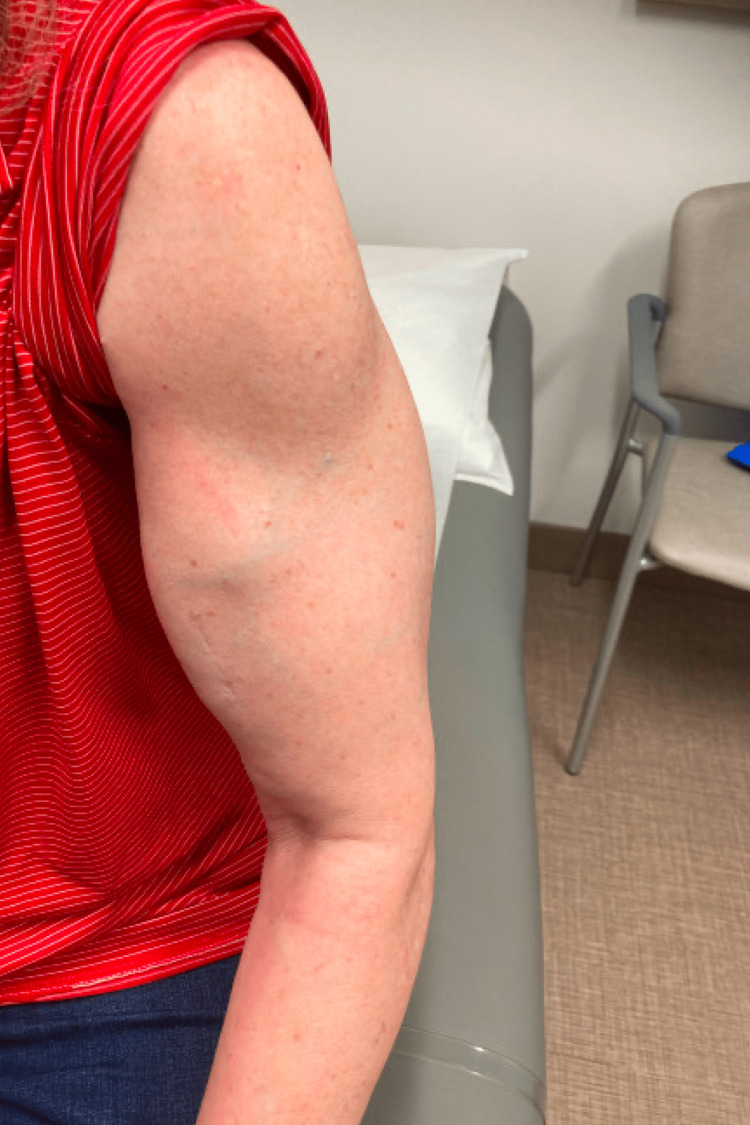
Left arm hypertrophy

**Figure 2:**
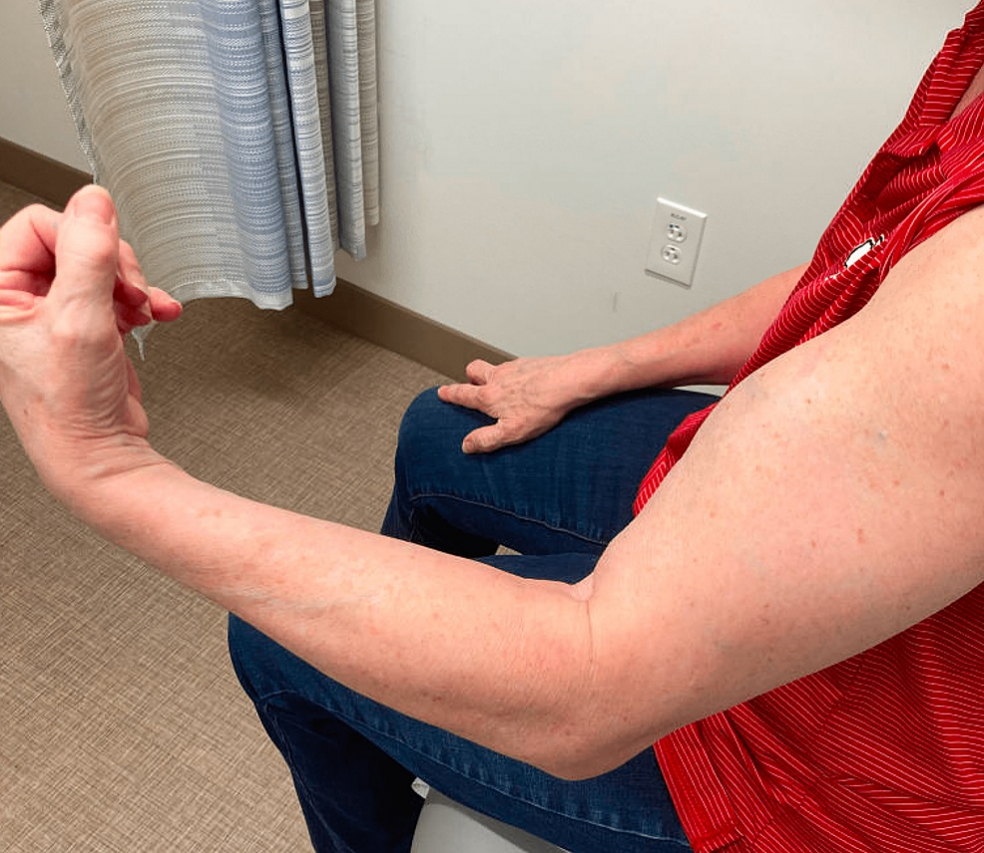
Maximum degree of flexion at the elbow

While on oral steroid therapy, she had improvement in her lower extremity weakness. She was started on mycophenolate mofetil, and the steroid dose was gradually lowered. Despite this, the patient had a persistent limited range of motion of the left upper extremity and increasing muscular hypertrophy in both arms. An MRI of the upper extremities showed hypertrophy and edema with enhancement of the musculature. At this time, she was referred to our clinic for further assessment. On exam, she had mild hip flexor weakness and marked hypertrophy of bilateral biceps (L>R) (Figure 1) with the inability to straighten the fingers of the right hand. She was unable to perform elbow flexion of the left arm beyond 150 degrees at the angle of the cubital fossa (Figure 2). There was also hypertrophy of the trapezius, deltoids, and triceps muscles. She had delayed relaxation in the left hand after voluntary contraction. The remainder of the neurological exam was normal.

**Figure 3:**
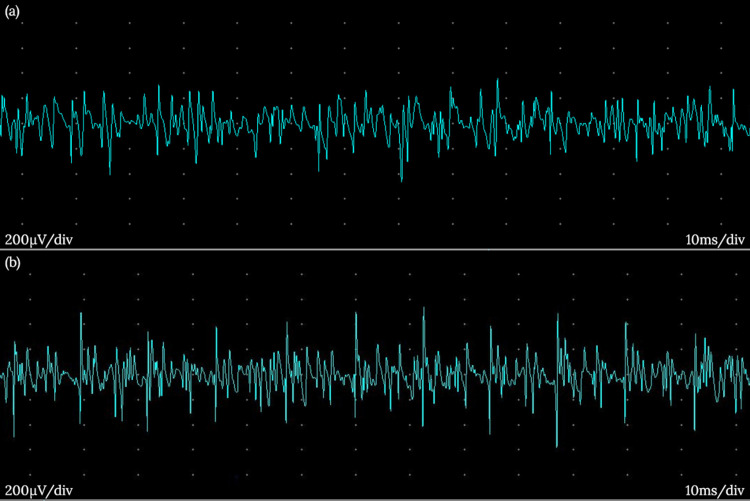
Needle EMG of the left biceps with neuromyotonia (a) and complex repetitive discharges (b) EMG: electromyography

A repeat myositis panel was positive for the Mi-2 antibody. Needle electromyography (EMG) of the bilateral biceps and right triceps muscles showed prominent neuromyotonia, myokymia, and complex repetitive discharges, as can be seen in Isaacs syndrome (Figure 3). Voltage-gated potassium channel (VGKC), leucine-rich glioma inactivated protein 1 (LGI-1), and contactin-associated protein 2 (CASPR2) antibodies were negative. The patient was started on carbamazepine with some improvement in her clinical stiffness.

## Discussion

Dermatomyositis is an inflammatory muscle disease that typically presents with symmetric proximal muscle weakness and cutaneous features such as Gottron’s papules and heliotropic rash. Muscle damage is thought to be a consequence of humoral-mediated autoimmunity against muscle micro-vasculature. Membrane attack complex deposition into vascular walls causes inflammatory changes and subsequent ischemic injury to muscle fibers [6,7]. Muscle biopsy will typically show perivascular and perimysial inflammatory infiltrates along with perifascicular atrophy. Several different autoantibodies are recognized in clinical diseases, some of which are tightly associated with malignancy [8].

Isaacs syndrome is an acquired immune-mediated disorder of peripheral nerve hyperexcitability (PNH). Clinically, this can manifest as cramps, delayed relaxation, fasciculations, myokymia, and muscular hypertrophy. Extra-muscular symptoms of the condition can include autonomic manifestations such as sweating and diarrhea. Nerve conduction studies can show after-discharges, and electromyography can reveal fasciculation potentials, as well as myokymic and neuromyotonic discharges. These discharges are typically present during sleep and throughout general anesthesia.

Thirty-eight percent to 50% of cases are associated with autoantibodies against VGKC complexes, including CASPR2 and LGI-1 antibodies [3,4]. A subset of patients have overlapping central nervous system involvement, such as in Morvan’s syndrome or limbic encephalitis [9]. There is some association with underlying malignancy in some patients, with small-cell lung cancer, thymoma, and Hodgkin lymphoma being the most associated.

Our patient had Mi-2 antibody-positive dermatomyositis with clinical and electromyographic evidence of Isaacs syndrome. She did not have autonomic features, central nervous system involvement, VGKC, LGI-1, or CASPR2 antibodies. Although peripheral nerve hyperexcitability was diagnosed later in her clinical course, it can be speculated to have been present early due to her reported symptoms of stiffness and limited range of motion in the upper extremities at the onset of her weakness and rash. An interesting feature of her examination was the marked muscle hypertrophy and significantly limited range of motion in her upper extremities. 

Although neuromyotonia can be seen in the presence of other autoimmune diseases, a rare association with dermatomyositis has been reported. A review of the literature shows two prior case reports of dermatomyositis with either peripheral nerve hyperexcitability or Isaacs syndrome - both of which were in patients either previously diagnosed or later diagnosed with systemic lupus erythematosus [10,11]. Neither of these patients was reported to be diagnosed with an underlying malignancy. Interestingly, similar to our patient, one was also Mi-2 antibody positive [10].

Mi-2 is a component of the nucleosome remodeling and histone deacetylase (NuRD) machinery and is affiliated with chromosomal transcriptional regulation [12]. It is felt to be a myositis-specific antibody and has not been associated with PNH. Within muscle, it is upregulated in regenerating muscle fibers [13]. The ongoing destruction and subsequent renewal of skeletal muscle cells in dermatomyositis could explain such an autoantibody. Unlike some other myositis-specific antibodies, Mi-2 antibodies have a relatively lower risk of underlying malignancy [14]. In our patient, evaluation for underlying malignancy has thus far been negative.

The origin of PNH along the motor unit is variable, depending on the etiology. In acquired immune-mediated pathology, it has been speculated to originate at the nerve terminal due to increased permeability of the blood-nerve barrier at the junction. It is conceivable that microvascular involvement, such as that in dermatomyositis, further increases this susceptibility, and subsequent muscle damage exposes the motor nerve terminal, creating fertile ground for an immunogenic response to nerve terminal proteins. Autoimmunity at the nerve terminal may explain why myasthenia gravis, an autoimmune disorder at the neuromuscular junction, is one of the more commonly associated autoimmune disorders with peripheral nerve hyperexcitability [1,15].

Treatment of PNH involves the use of membrane-stabilizing medications, such as phenytoin and carbamazepine, which can treat clinical symptoms of cramps and stiffness [1,16]. Immunotherapy, including plasmapheresis, intravenous immunoglobulin (IVIG), prednisone, and other immunosuppressive medications, can help those with confirmed autoimmune etiologies [1].

## Conclusions

While muscle hypertrophy is not a typical feature of dermatomyositis, one must consider PNH or Isaacs syndrome when patients present with muscle stiffness and hypertrophy. Needle EMG testing will reveal marked abnormal spontaneous activity in the form of neuromyotonia, myokymia, and complex repetitive discharges. While serological testing for VGKC complex antibodies confirms Isaacs syndrome, some patients are antibody negative. Immunosuppressive therapy can help improve the disease state. Additionally, symptoms of Isaacs syndrome may respond to membrane-stabilizing drugs. Given the risk of malignancy in dermatomyositis and Isaacs syndrome, it is critical to screen for malignancy.
